# Imaging of intracellular fatty acids by scanning X-ray fluorescence microscopy

**DOI:** 10.1096/fj.201600569R

**Published:** 2016-09-06

**Authors:** Mari Shimura, Hideo Shindou, Lukasz Szyrwiel, Suzumi M. Tokuoka, Fumie Hamano, Satoshi Matsuyama, Mayumi Okamoto, Akihiro Matsunaga, Yoshihiro Kita, Yukihito Ishizaka, Kazuto Yamauchi, Yoshiki Kohmura, Ryszard Lobinski, Isao Shimizu, Takao Shimizu

**Affiliations:** * Department of Intractable Diseases National Center for Global Health and Medicine, Tokyo, Japan;; † Riken SPring-8 Center, Hyogo, Japan;; ‡ Lipid Signaling, Research Institute, National Center for Global Health and Medicine, Tokyo Japan;; § Agency for Medical Research and Development–Core Research for Evolutionary Science and Technology (AMED–CREST), Tokyo, Japan;; ¶ Centre National de la Recherche Scientifique/University of Pau and Pays de l’Adour (CNRS/UPPA), Laboratoire de Chimie Analytique, Bio-Inorganique et Environnement (LCABIE), Unité Mixte de Recherche 5254, Pau, France;; ‖ Department of Chemistry of Drugs, Wroclaw Medical University, Wroclaw, Poland;; # Department of Lipidomics, Graduate School of Medicine, The University of Tokyo, Tokyo, Japan;; ** Life Sciences Core Facility, Graduate School of Medicine, The University of Tokyo, Tokyo, Japan;; †† Department of Precision Science and Technology, Graduate School of Engineering, Osaka University, Osaka, Japan; and; ‡‡ Research Institute for Science and Engineering, School of Advanced Science and Engineering, Waseda University, Tokyo, Japan

**Keywords:** glycerophospholipid, labeling, cellular membrane, lipid metabolism, visualization

## Abstract

Fatty acids are taken up by cells and incorporated into complex lipids such as neutral lipids and glycerophospholipids. Glycerophospholipids are major constituents of cellular membranes. More than 1000 molecular species of glycerophospholipids differ in their polar head groups and fatty acid compositions. They are related to cellular functions and diseases and have been well analyzed by mass spectrometry. However, intracellular imaging of fatty acids and glycerophospholipids has not been successful due to insufficient resolution using conventional methods. Here, we developed a method for labeling fatty acids with bromine (Br) and applied scanning X-ray fluorescence microscopy (SXFM) to obtain intracellular Br mapping data with submicrometer resolution. Mass spectrometry showed that cells took up Br-labeled fatty acids and metabolized them mainly into glycerophospholipids in CHO cells. Most Br signals observed by SXFM were in the perinuclear region. Higher resolution revealed a spot-like distribution of Br in the cytoplasm. The current method enabled successful visualization of intracellular Br-labeled fatty acids. Single-element labeling combined with SXFM technology facilitates the intracellular imaging of fatty acids, which provides a new tool to determine dynamic changes in fatty acids and their derivatives at the single-cell level.—Shimura, M., Shindou, H., Szyrwiel, L., Tokuoka, S. M., Hamano, F., Matsuyama, S., Okamoto, M., Matsunaga, A., Kita, Y., Ishizaka, Y., Yamauchi, K., Kohmura, Y., Lobinski, R., Shimizu, I., Shimizu, T. Imaging of intracellular fatty acids by scanning X-ray fluorescence microscopy.

When fatty acids are taken up by cells, they move around in the cytosol with fatty acid binding proteins and are then converted to acyl-CoAs (1, 2). Acyl-CoAs are common intermediates for further metabolism, such as β-oxidation, elongation, desaturation, or esterification with glycerol or glycerol 3-phosphate, for the biosynthesis of triacylglycerol or glycerophospholipids (3–9). Thus, fatty acids are efficient energy sources by themselves or are components of lipid droplets or cellular membranes (6, 10, 11). Such versatile functions of fatty acids are essential for cell survival, growth, and movement (10, 11). Although the metabolic tracing of fatty acids and their derivatives by biochemical approaches has been widely performed, little information is available on the intracellular localization and dynamic movement of fatty acids within cells. Chromophore labeling is often used, but labeling with large molecules may interfere with metabolism by steric hindrance (12). Isotope labeling of fatty acids in combination with positron emission tomography (PET) is a promising approach (13). Matrix-assisted laser desorption ionization or desorption electrospray ionization mass spectrometry is an effective procedure that does not require labeling (14). However, both technologies are inadequate to determine the intracellular localization of fatty acids due to their low resolution (>5 μm).

To overcome this issue, we used scanning X-ray fluorescence microscopy (SXFM), which enables the imaging of multiple intracellular elements at the suborganelle level by the combination of a synchrotron radiation source and a sub-100-nm X-ray beam focusing system (15, 16). Using this approach, we have observed intracellular trace elements, including multiple elements that are essential for cellular maintenance and nonessential elements such as platinum from anticancer drugs (17, 18). In this study, we applied SXFM for the imaging of intracellular fatty acids and their derivatives. Single-element labeling combined with SXFM technology facilitates the process, which may provide new clues for determining dynamic changes in fatty acids at the single-cell level. Here, we aimed to visualize the incorporation and localization of palmitic acid (PA, C16:0) and stearic acid (SA, C18:0) by Br labeling.

## MATERIALS AND METHODS

### Chemical synthesis

All reagents and solvents were purchased commercially and used as received. ^1^H nuclear magnetic resonance (NMR) and ^13^C NMR spectra were recorded on a spectrometer (AL-400; Jeol, Akishima, Japan) using CDCl_3_ as the solvent and tetramethylsilane as an internal standard. Multiplicities are indicated as br (broadened), s (singlet), d (doublet), t (triplet), q (quartet), and m (multiplet). Infrared (IR) spectra were recorded on a JIR-WINSPECK 50 FT-IR spectrometer (Jeol), and *V*_max_ values are presented in reciprocal centimeters. Bands were characterized as br (broad), s (strong), m (medium), or w (weak). High-resolution mass spectrometry (HRMS) spectra were recorded (JMS-SX102A; Jeol). The purity of all isolated materials was demonstrated by NMR (free of obvious impurities) and thin-layer chromatography (homogeneous material). For 12-bromopalmitic acid, a stirred solution of 12-hydroxyhexadecanoic acid (0.127 g, 0.47 mmol), PPh_3_ (0.260 g, 0.99 mmol), and imidazole (0.068 g, 1.0 mmol) in CH_2_Cl_2_ (6.5 ml) was added slowly to a solution of CBr_4_ (0.329 g, 0.99 mmol) in CH_2_Cl_2_ (1 ml) at 0°C. The mixture was stirred at 0°C to room temperature for 4 h and quenched with 1 N aqueous HCl. The aqueous solution was extracted with ethyl acetate (EtOAc), and the combined organic phase was washed with brine and dried over anhydrous Na_2_SO_4_. After removal of the solution *in vacuo*, the residue was purified by silica gel column chromatography using EtOAc/hexane (5:95) to give 12-bromopalmitic acid (0.055 g, 35%) as a white solid. The following results were obtained: ^1^H NMR (400 MHz, CDCl_3_): δ0.92 (t, 3H, *J* = 7.3 Hz), 1.28 to 1.63 (brs, 20H, 1.80 (m, 4H), 2.35 (t, 2H, *J* = 7.3 Hz), 4.03 (m, 1H); ^13^C NMR (100 MHz, CDCl_3_): δ 13.9, 22.1, 24.6, 27.5, 29.0, 29.1, 29.3, 29.4, 29.7, 34.0, 38.8, 39.1, 58.9, and 180.1; IR (neat, ATR): 2914, 2851, 1697, 1471, 1428, 1408, 1270, 1210, 918, and 717 cm^−1^; and HRMS (ESI) *m/z* calculated for C_16_H_31_BrO_2_Na[M + Na]: 357.1405, found [M + Na]: 357.1397. For 12-bromostearic acid, a stirred solution of 12-hydroxyoctadecanoic acid (0.150 g, 0.50 mmol), PPh_3_ (0.526 g, 2.0 mmol), and imidazole (0.136 g, 2.0 mmol) in CH_2_Cl_2_ (3.0 ml) was added slowly to a solution of CBr_4_ (0.664 g, 2.0 mmol) in CH_2_Cl_2_ (2 ml) at 0°C. Then, the mixture was stirred at 0°C to room temperature for 21 h and quenched with 1 N aqueous HCl. The aqueous solution was extracted with EtOAc, and the combined organic phase was washed with brine and dried over anhydrous Na_2_SO_4_. After removal of the solution *in vacuo*, the residue was purified by silica gel column chromatography using EtOAc/hexane (5:95) to give 12-bromostearic acid (0.129 g, 71%) as a white solid. The following results were obtained: ^1^H NMR (400 MHz, CDCl_3_): δ0.89 (t, 3H, *J* = 7.3 Hz), 1.28 to 1.63 (brs, 25H), 1.80 (m, 4H), 2.35 (t, 2 H, *J* = 7.3 Hz), 4.03 (m, 1H); ^13^C NMR (100 MHz, CDCl_3_): δ14.0, 22.5, 24.6, 27.5, 28.7, 29.0, 29.1, 29.3, 29.4, 31.6, 33.9, 39.1, 59.0, and 179.3; IR (neat, ATR): 2952, 2914, 2851, 1697, 1471, 1428, 1408, 1270, 1210, 918, and 717 cm^−1^; and HRMS (ESI) *m/z* calculated for C_18_H_35_BrO_2_Na[M + Na]: 385.1718, found [M + Na]: 385.1712.

Each amount of synthesized Br-PA and Br-SA was determined by gas chromatography with a flame ionization detector (GC-2010Plus AF; Shimadzu, Kyoto, Japan) using PA and SA as standards, respectively.

### Cell treatment

The RBRC-RCB02859 CHO-K1 cell line was obtained from the Riken Cell Bank of Japan (Tsukuba, Japan). Cells were maintained in Ham’s F-12 medium (Nakalai Tasque, Kyoto, Japan) supplemented with 10% heat-inactivated fetal calf serum (104387; Gibco, Grand Island, NY, USA). Cells (2 × 10^5^ per 6-cm dish) were plated 21 h before treatment. Br-labeled fatty acids (3.2 µM Br-PA or 2.1 µM Br-SA) were incubated at 37°C. After treatment, the cells were washed twice with PBS(−) (P-4417; Sigma-Aldrich, St. Louis, MO, USA) containing 10% FBS and then once with PBS(−). For inductively coupled plasma mass spectrometry (ICP-MS) and liquid chromatography mass spectrometry (LC-MS) analyses, cells were collected using a scraper (3008; Corning, Corning, NY, USA) or extracted using MeOH. The cell pellet and extract were collected by centrifugation at 800 and 10,000 *g* at 5°C, respectively.

### SXFM

Cells were plated on a 200-nm-thick SiN membrane (NTT Advanced Technology, Atsugi, Japan) 21 h before treatment. After treatment, the cells were washed twice with PBS(−) containing 10% FBS and then once with PBS(−). The cells were fixed with 2% paraformaldehyde (18814, ultra-pure EM grade; Polysciences, Inc., Warrington, PA, USA) in PBS(−) for 10 min. The plates were washed with PBS(−) once and Milli-Q water 3 times, air-dried, and stored in clean tubes. Differential interference contrast images were obtained by an Olympus BX51 microscope (Melville, NY, USA) equipped with a Plan Apochromat objective lens. TIFF images acquired using SPOT Advanced (Diagnostic Instruments, Inc.). SXFM was performed using the undulator beamline BL29XU of the SPring-8 synchrotron radiation facility by combining a Kirkpatrick-Baez type X-ray focusing system, an *xy*-scanning stage for scanning the sample mounting, and an energy dispersive X-ray detector (Vortex-90EX; Hitachi High-Technologies Science America, Inc., Northridge, CA, USA). Monochromatic X-rays at 15 keV were focused down to 500 × 500 nm^2^ for a large area scan and to 250 × 250 nm^2^ for a high-resolution scan. The typical photon flux for the 500-nm beam is approximately 2 × 10^11^ photons/s. The X-ray fluorescence spectrum was recorded using a 1 s exposure for each pixel. The fluorescence signals of each element of interest were extracted and normalized based on the incident beam intensity. After scanning the whole area, elemental distributions were visualized digitally. SXFM produced superimposed signals from the samples in the vertical direction. In addition to the mapping images, the element concentration per area (µm^2^) was analyzed quantitatively using thin nickel and platinum films, the thickness and density of which were decided in advance. The Br signal intensities per cellular area in the TIFF images and their surface plot images were acquired using ImageJ software (National Institutes of Health, Bethesda, MD, USA). For fluorescence dye application combined with SXFM, the mitochondria marker MitoTracker green (1:10,000, M7512; Invitrogen) was applied to living cells for 20 min at 37°C after the Br-labeled fatty acids treatment. The cells were fixed with 2% paraformaldehyde (18814, ultra-pure EM grade; Polysciences, Inc., Warrington, PA, USA) in PBS(−) for 10 min. To observe the endoplasmic reticulum (ER), 4.3 nM of DiOC6 (3) (Thermo Fisher Scientific, Waltham, MA, USA) was applied to the cells for 15 min at room temperature after the Br-labeled fatty acids treatment and the fixation. The plates were replaced with fresh PBS(−). Cells were observed under a microscope (IX70; Olympus, Melville, NY, USA) equipped with a LCPlanFI microscope objective lens and a SPOT Pursuit CCD camera (Diagnostic Instrument Inc., Sterling Heights, MI, USA). After the fluorescence observation, cells were washed with Milli-Q water 3 times and air-dried for SXFM measurement. TIFF images acquired using Spot Advanced (Diagnostic Instruments, Inc.) were imported into Photoshop (Adobe Systems, San Jose, CA, USA).

### Separation of lipid classes

Cellular lipids extracted using MeOH were evaporated and dissolved in 1 ml of chloroform and then applied to aminopropyl cartridges (200 mg, InertSep NH2; GL Sciences Inc., Tokyo, Japan) preconditioned with hexane. Neutral lipid, free fatty acid, and phospholipid (mainly PC and PE) fractions were obtained by sequential elution using 10 ml of chloroform/isopropanol (2:1, v/v), 2% acetic acid in diethyl ether, and methanol, respectively. Flow-through fractions were combined with the chloroform/isopropanol (2:1) eluent as the neutral lipid fraction. The fractionated samples were evaporated and used for the detection of Br signals by ICP-MS.

### ICP-MS

The concentrations of ^79^Br were measured using an Agilent 7500 or 7700 ICP-MS instrument fitted with Pt cones and a MicroMist nebulizer and optimized for ^79^Br sensitivity. The measured ^79^Br/^81^Br isotope ratio corresponded to the natural value. Standards and washing solutions were prepared in 0.5% tetramethylammonium hydroxide (TMAH) (TraceSelect; Sigma-Aldrich). Samples containing 2 × 10^5^ to 3 × 10^6^ cells were placed in Eppendorf tubes as described in the preceding section. Then, 79 µl of 25% TMAH were added, and the tube was closed tightly and heated for 3 h at 60°C. No difference in mass before and after heating was observed. After cooling, the sample was transferred to a 5 ml tube. The tube was rinsed with 0.5 ml of water, and the original and wash liquids were combined and brought up to a 4 ml volume with water. A blank was prepared with the samples. The detection limit was calculated as 5 standard deviations of the blank (0.3 µg/L). The average relative standard deviation was 2.1% (range, 1.7–5%). A quality-control sample, prepared by mixing the standard with 0.5% TMAH, was analyzed after every 10 samples. Quantification was performed using a 5- to 100-ppb standard curve, which covered the range of Br concentrations observed in the samples (samples containing higher Br concentrations were diluted). The method was validated by the determination of Br in Seronorm Trace Element Serum. Analyses of a second series of cells and their insoluble fractions (fatty acid, neutral lipids, and glycerophospholipids) were performed using the Agilent 7700 instrument optimized for ^79^Br. The measured ^79^Br/^81^Br isotope ratio corresponded to the natural value. The preparation of fractions from MeOH extracts is described in the preceding section. TMAH (79 µl) was added to Eppendorf tubes containing dry samples, followed by heating for 3 h at 60°C. The samples were transferred to 5-ml tubes by washing the Eppendorf tubes 3 times with 0.5 ml of 90% methanol/water (Chromasolv; Sigma-Aldrich). Water-soluble fractions were prepared as previously described.

### Liquid chromatography-selected reaction monitoring-mass spectrometry

Liquid chromatography-selected reaction monitoring-mass spectrometry was performed using the Nexera Ultra-High Performance Liquid Chromatography (UHPLC) system and LC-MS-8040 triple quadrupole mass spectrometer (Shimadzu Corp., Kyoto, Japan) (19). A reverse-phase Acquity UPLC BEH C8 column (1.7 μm, 2.1 × 100 mm) (Waters, Milford, MA, USA) was used with a ternary mobile phase system. For mobile phases A, B, and C, 5 mM NH_4_HCO_3_/water, acetonitrile, and isopropanol were used, respectively, with the following linear gradient [time (%A/%B/%C)]: 0 min (75/20/5) to 20 min (20/75/5) to 40 min (20/5/75) to 45 min (5/5/90) to 50 min (5/5/90) to 55 min (75/20/5). The flow rate was 0.35 ml/min. The oven temperature was set at 47°C. The injection volume was 5 µl. The following transitions were monitored: [M + H]^+^ → 184 for unlabeled PC, [M + 81]^+^ → 184 for ^81^Br-labeled PC, and [M + 79]^+^ → 184 for ^79^Br-labeled PC. The chromatogram peaks of ^81^Br-labeled PC and ^79^Br-labeled PC exhibited similar signal intensities at the same chromatogram retention time, which corresponded to the natural abundances of ^81^Br and ^79^Br. Major Br-labeled PC species detected in Br-PA- or Br-SA-treated cells were PC30:0, 32:0, 32:1, 34:0, 34:1, 36:0, 36:1, and 36:2 (Supplemental Table 1). Accordingly, ^81^Br-labeled PC peak areas among the PC species in Supplemental Table 1 are shown as representative Br-labeled PC.

### Gas chromatography with electron impact mass spectrometry

Gas chromatography with electron impact mass spectrometry was carried out using a GCMS-QP2010 Ultra (Shimadzu, Kyoto, Japan). A FAMEWAX capillary column (30 m × 0.25 mm I.D. × 0.25 μm) (Restek; Shimadzu) was used for fatty acid methyl ester analysis. The injection port temperature was set at 250°C. A 1-μl aliquot was injected in splitless mode. The column temperature was programmed as follows: the initial temperature was held at 40°C for 2 min; increased at 20 to 140°C/min, 11 to 200°C/min, and 3°C to 240°C/min; and then maintained at 240°C for 10 min. Helium was used as the carrier gas with a linear velocity of 45 cm/s. Methyl esters of Br-fatty acids were detected in the selected ion monitoring mode of the characteristic fragments (*m/z* 269 for Br-PA and *m/z* 297 for Br-SA). Quantification was done by the internal standard method using *n*-tricosanoic acid (C23:0, Sigma-Aldrich; selected ion monitoring *m/z* 368) as an internal standard. The concentrations of the unknown samples were determined using calibration curves prepared by analyzing known concentrations of methyl esters for Br-PA, Br-SA, and *n*-tricosanoic acid.

## RESULTS

**Figure 1.**
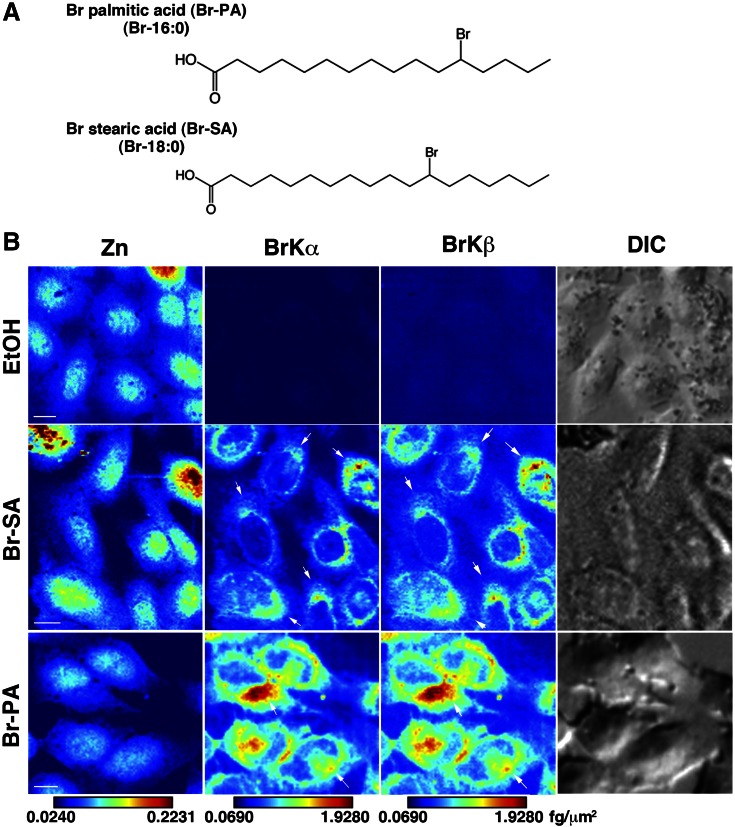
X-ray fluorescence images of Br-labeled fatty acids. *A*) Schematic structure of the Br-labeled fatty acids. *B*) Zn- and Br-mapping images of Br-SA-, Br-PA-, and EtOH-treated CHO-K1 cells. Cells were treated with 2.1 µM Br-SA and 3.2 µM Br-PA for 24 h. Arrows indicate the Br signals, which tended to be clustered. Zn, zinc; BrKα and Brkβ, bromine X-ray emission lines; DIC, differential interference contrast image. Br-SA, cells treated with Br-labeled stearic acid; Br-PA, cells treated with Br-labeled palmitic acid. A brighter color indicates higher signal intensity. Color bar, femtograms per square micrometer; scale bar, 10 μm.

We first prepared Br-PA and Br-SA, which possess Br at position ∆12 (see Materials and Methods) (**Fig. 1*A***). Cells were treated with different concentrations of Br-SA for 24 h and subjected to SXFM. The Br-SA SXFM spectrum of cells showed that the Br X-ray fluorescence lines with different electron shells (Br-Kα and Br-Kβ) increased in a manner dependent on the Br-SA concentration, whereas for the rest of the elements, including zinc (Zn), X-ray fluorescence energy (Zn-Kα and Zn-Kβ signals) was relatively stable (Supplemental Fig. 1). Whereas untreated control cells showed background Br signals due to the use of serum-containing culture medium, Br-SA-treated cells exhibited more than 10-fold higher Br contents, which enabled mapping images ready for SXFM. Spectra of Br-Kα and Zn-Kα were mainly used for mapping images (Supplemental Fig. 1, arrows). We next treated cells with the same volume of Br-SA and Br-PA at concentrations of 2.1 and 3.2 µM, respectively, as estimated by gas chromatography (see Materials and Methods) and subjected them to SXFM. Br-Kα and Br-Kβ signals were similarly found in cells treated with Br-fatty acids, whereas the signals did not exceed the background levels in ethanol (EtOH)-treated cells (Fig. 1*B*). In both Br-SA- and Br-PA-treated cells, Br signals were detected mostly in the perinuclear region of the cytoplasm and to a lesser extent in the nuclear region. The Br signals tended to be clustered on one side of the nucleus (Fig. 1*B*, arrows). In contrast, Zn signals were observed in the entire cellular region but were mainly enriched in the nucleus, similar to previous findings (18). The signals were analyzed semiquantitatively using a standard curve, and the intensities were illustrated as a color bar (see Materials and Methods). The signals of Br-PA were approximately 2-fold greater than those of Br-SA (Fig. 1*B*), due at least in part to the different concentrations of Br-fatty acids added to the culture medium.

**Figure 2.**
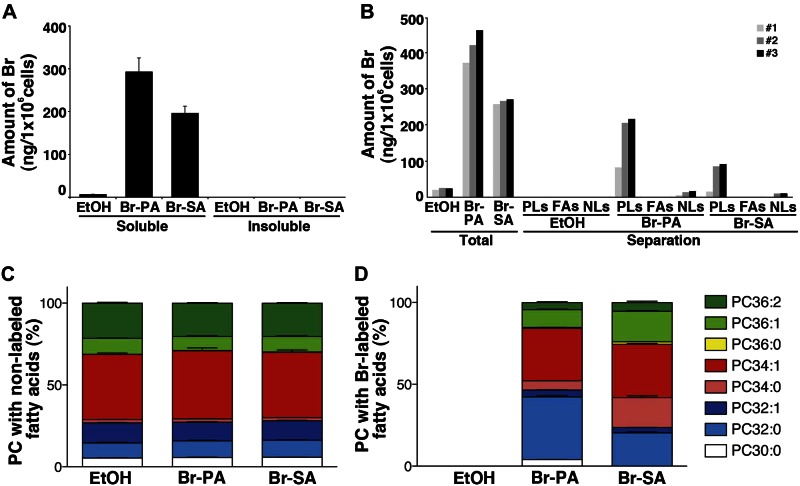
ICP-MS measurement and LC-MS analyses of the cells under the same conditions as in Fig. 1. *A*) The Br contents of MeOH-soluble extracts and insoluble pellet fractions were measured by ICP-MS. Data in the insoluble pellet fractions were all lower than the limit of quantitation. *B*) The Br contents in the lipids. MeOH extracts were separated into glycerophospholipids (PLs), fatty acids (FAs), and neutral lipids (NLs). “Total” indicates the cell extracts before separation. Three independent experiments were performed and are presented as *1*–*3*. The values after extraction from EtOH-treated cells were determined, and all values in FAs were lower than the limit of quantitation. *C*) Distribution of the proportions of unlabeled or Br-labeled PC species; the 8 major PC species indicated were measured by LC-MS. Stacked bar graphs displaying the distribution of PC species with unlabeled and Br-labeled fatty acids; 100% on the *y* axis indicates the sum of each PC peak area. The data were normalized to the number of cells. Data are given as means + sem (*n* = 4). Br, bromine Br-PA, cells treated with Br-labeled palmitic acid; Br-SA, cells treated with Br-labeled stearic acid; EtOH, EtOH-treated cells; PC, phosphatidylcholine.

We then measured the intracellular Br levels using ICP-MS. The intensity of the Br signal from Br-PA-treated cells in the soluble methanol (MeOH) fraction was higher than that from Br-SA-treated cells (**Fig. 2*A***), whereas the Br signal did not exceed the background levels in EtOH-treated cells, in agreement with our imaging findings (Fig. 1*B*). On the other hand, the level of Br was lower than the limit of quantitation in the MeOH-insoluble pellets (Fig. 2*A*). These findings suggest that the major Br-labeled fatty acids were present in the lipid fraction (soluble MeOH) but not in the protein fraction (insoluble pellets). We next examined the distribution of Br-labeled fatty acids in lipids. MeOH extracts from cells were separated using aminopropyl cartridges (see Materials and Methods) and processed for ICP-MS (Fig. 2*B*). Glycerophospholipids were the major components detected, whereas lower levels of neutral lipids were found; the levels of free fatty acids were lower than the limit of quantitation for both Br-PA and Br-SA. We next analyzed the composition of phosphatidylcholine (PC), one of the major components of glycerophospholipids, with LC-MS (Fig. 2*C*). The ratio of the PC composition using unlabeled fatty acids was unaffected by the addition of Br-labeled fatty acids (Fig. 2*C*, left). Several species of Br-labeled PCs (Br-PCs) were detected by the addition of Br-PA and Br-SA to the culture medium (Fig. 2*C*, right). Br-PC32:0 and Br-PC34:1 were the major Br-PCs detected upon the addition of Br-PA and Br-SA, respectively (Fig. 2*C*, right). To determine the fate of Br-labeled fatty acids after incubation, cells treated with Br-labeled fatty acids were subjected to gas chromatography. From the analysis, it is notable that Br-SA was detected as Br-PA and Br-SA in the cells, although most Br-PA was present mainly as Br-PA (Supplemental Fig. 2). These findings suggest that Br-PA and Br-SA were metabolized differently.

**Figure 3.**
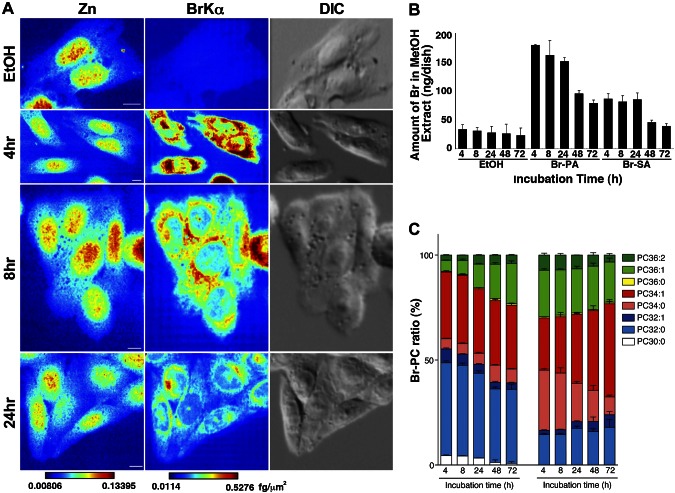
X-ray fluorescence images of cells with pulse labeling of Br-labeled fatty acids. *A*) Zn- and BrKα-mapping images of CHO-K1 cells pulse labeled for 4 h with 3.2 µM Br-PA followed by washing. BrKα, a bromine X-ray emission line; DIC, differential interference contrast image; Zn, zinc. A brighter color indicates higher signal intensity. Color bar, femtograms per square micrometer; scale bar, 10 μm. *B*) ICP-MS measurement analyses of cells pulse labeled with Br-labeled fatty acids. CHO-K1 cells pulse-labeled for 4 h with 3.2 µM Br-PA or 2.1 µM Br-SA followed by washing. Br contents of MeOH extracts according to incubation time after 4 h of pulse labeling with Br-labeled fatty acids. *C*) Percent ratios of Br-PC; the 8 major PC species indicated were measured by LC-MS; 100% on the *y* axis indicates the sum of each PC peak area. Data were obtained from 3 independent experiments. Data are given as means + sem (*n* = 3). Br-PA, cells treated with Br-labeled palmitic acid; Br-SA, cells treated with Br-labeled stearic acid; EtOH, EtOH-treated cells; PC, phosphatidylcholine.

We next pulse-labeled cells with Br-PA (3.2 µM) and Br-SA (2.1 µM) for 4 h, followed by washing. Br signals were abundant in the perinuclear region 4 h after pulse labeling with Br-PA; they then decreased at 8 and 24 h, although the strongest signals remained in the perinuclear region (**Fig. 3*A***). The Br contents of MeOH extracts from cells pulse-labeled with Br-PA or Br-SA decreased, especially after 24 h (Fig. 3*B*), when cells underwent contact inhibition (Supplemental Fig. 3). We observed that the fraction of PC36:1 increased during a 72-h incubation in Br-PA-treated cells, whereas the fraction of PC34:0 decreased and that of PC34:1 increased in Br-SA-treated cells (Fig. 3*C*). These MS data show that Br-PA and Br-SA were metabolized over time.

**Figure 4.**
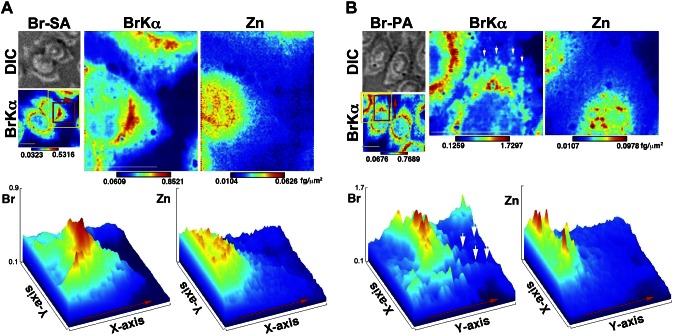
Higher-resolution X-ray fluorescence images. *A*) Top: Br-SA. Bottom: a surface plot generated based on the red area in the top images. *B*) Top: Br-PA. Bottom: a surface plot generated based on the red area in the top images. The yellow-framed area was measured at a higher resolution. Red arrows indicate the direction presented in the surface plots; white arrows indicate the spot-like Br distribution. BrKα, a bromine X-ray emission line; Br-SA, cells treated with Br-labeled stearic acid; Br-PA, cells treated with Br-labeled palmitic acid; DIC, differential interference contrast image; Zn, zinc. A brighter color indicates higher signal intensity. Color bar, femtograms per sqare micrometer; scale bar, 10 μm.

These findings encouraged us to obtain higher-resolution images to assess the Br distribution in cells. Strong Br signals were detected in the perinuclear region (**Fig. 4*A***). On the other hand, Zn signals were localized mainly in the nuclei, suggesting the distinctiveness of the Br localization signals (Fig. 4*A*, bottom and surface plots). Interestingly, some cells showed spot-like Br signals in the cytoplasm, which were distinct from the Zn signals (Fig. 4*B*, arrows and surface plots). Eleven percent of Br-SA- and 45% of Br-PA-treated cells showed spot-like Br signals for 24 h (Supplemental Fig. 4*A*, *B*). A spot-like distribution was also observed 24 h after pulse labeling (Fig. 3*A*). Numerous smaller multiple spots were revealed rather than continuous labeling, which was confirmed by an independent experiment (Supplemental Fig. 4*C*).

**Figure 5.**
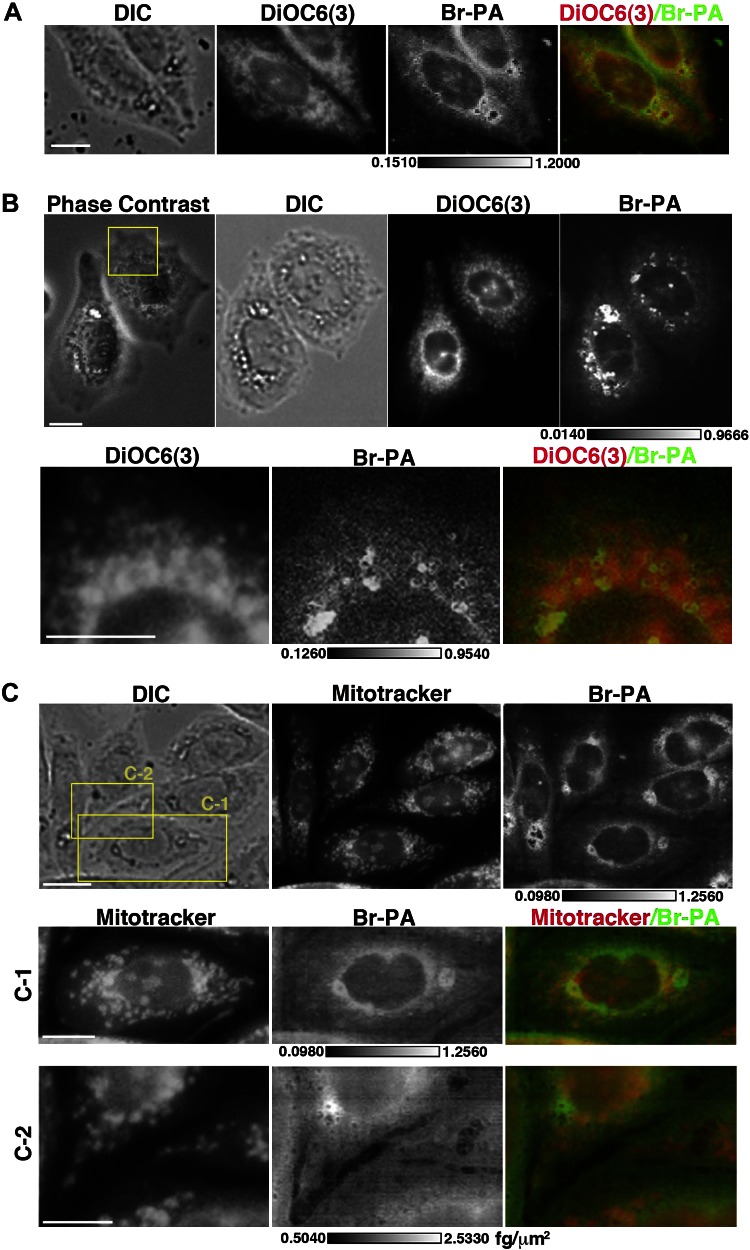
Colocalization of Br signals and DiOC6(3) and MitoTracker. *A*, *B*) Comparison between DiOC6(3) fluorescence image and Br signals from SXFM (500 nm/pixel). *B*) The area framed in yellow in the top image was observed using higher-resolution SXFM (250 nm/pixel) (bottom). *C*) Comparison between MitoTracker green fluorescence image and Br signals from SXFM. Yellow-framed area in the top image (C-1, C-2) was observed by higher resolution SXFM (250 nm/pixel) (bottom). DIC, differential interference contrast images; DiOC6(3) or MitoTracker fluorescence dye signals; Br-PA, SXFM bromine signals from BrKα; phase-contrast, phase-contrast images. Merged image, red, DiOC6(3) or MitoTracker; green, bromine; scale bar, 10 μm; bar in bromine, femtograms per square micrometer.

We next imaged cells stained with a mitochondrial marker (Mitotracker) and an ER marker (DiOC6) (3) dyes before SXFM imaging (**Fig. 5**). Because these dyes did not affect the X-ray energy spectrum, with the exception of a small increase in copper (data not shown), we compared the dye and bromine images using SXFM. The data suggest that Br signals were similar to those of DiOC6 (3), particularly in the perinuclear region (Fig. 5*A*, *B*), but did not always label mitochondria (Fig. 5*C*).

## DISCUSSION

In this study, intracellular Br-labeled fatty acids were successfully visualized at the single-cell level. Our findings suggest that single-element labeling of small molecules combined with SXFM technology facilitates the intracellular imaging of fatty acids and their derivatives, including phospholipids.

### SXFM measurements on Br-lipids

The SXFM shows high resolution and high sensitivity for imaging the elemental distribution compared with that of Raman microscopy and mass spectrometry imaging (20, 21). On the other hand, Raman microscopy is promising for imaging living cells with labels or unlabeled fatty acids (20). Raman microscopy and mass spectrometry imaging are commercially available, whereas SXFM is limited due to the need for a synchrotron beam. Therefore, complementary use of these methods will enable us to overcome practical issues. The SXFM resolution was 48 × 36 nm, which depended on the X-ray focusing technology. A 250-nm beam-sized image was more informative than 500 nm, which showed a spot-like distribution in this study. A smaller beam size, such as 70 nm, has been used to image mitochondria (18). We also produced a 7- × 7-nm focused beam with a multilayer mirror (22), which will be used to test biologic samples in a future study. The dynamic range of bromine by SXFM in this study was 0.0013 to 10 fg/µm^2^. The lower limit was due to Br background from the culture medium. Because the maximum bromine signals of cells were about 1 fg/µm^2^ in this study, our measurements were within the linear range and suitable for semiquantitative evaluation.

### The Br signals were mainly from glycerophospholipids

The Br signals were obtained without any major cytotoxicity and ER stress (data not shown). The Br signals detected by SXFM were mainly from glycerophospholipids, based on the column separation analysis. Br signals from Br-PA and Br-SA were located in the perinuclear region, suggesting a possible relationship with the ER and Golgi apparatus because the ER and Golgi are reportedly enriched with glycerophospholipid-related enzymes and related proteins (6, 10, 11, 23, 24). Staining with a mitochondrial or ER marker dye combined with SXFM measurements suggested that Br-PA colocalized with the ER in the perinuclear region more than in mitochondria, although we might not have determined the exact colocalization because dry and wet samples were compared. Using marker dyes containing unique metals or flash freezing and developing a cryoSXFM system would solve this issue.

We found a spot-like distribution at 24 h and in the high-resolution Br-PA images. A spot-like distribution was not always detected in Br-SA- and Br-PA-treated cells, suggesting that it might be related to cellular status (*e.g*., cell cycle, metabolism, *etc*.). Interestingly, a pulse-labeling study revealed numerous smaller spot-like distributions rather than continuous labeling. The high lipid content in the perinuclear region after continuous labeling may have hidden the smaller spot-like distribution. This may have reflected glycerophospholipids in the membranes of organelles such as mitochondria, peroxisomes, and lysosomes. Recently, multivesicular endosome (MVE) fusion with the plasma membrane and exosome release have been reported (25). The lipid content of MVEs is unclear, although glycerophospholipids and cholesterol are present (10, 25). The spot-like distribution of Br-labeled fatty acids also suggests the possibility of the presence of MVE-like structures in the cytosol. Additional studies using a larger number of samples under various culture conditions and stimuli would provide us with biologic information about the distribution of Br signals.

### Br-PA and Br-SA metabolism into PC

Br-labeled fatty acids can be activated to form acyl-CoA as a substrate for lysophospholipid acyltransferases, which biosynthesize glycerophospholipids using acyl-CoA (6), because Br-PA and Br-SA are converted mainly to glycerophospholipids. Several PC species containing Br-labeled fatty acids were detected by LC-MS. The addition of Br-PA resulted in Br-PC32:0 as the major product, possibly by the action of LPCAT1, as we demonstrated recently (26), and the level of Br-PC36:1 increased gradually. Br-SA affected mainly Br-PC34:0 but also increased the level of Br-PC34:1 in a time-dependent manner. These results suggest 2 possibilities: *1*) alterations occur in the fatty acid counterparts of Br-PA and Br-SA in PC, and *2*) the metabolism of Br-PA and Br-SA occurs by elongation, β-oxidation, or desaturation. These Br-labeled fatty acids should facilitate biochemical studies on lipid metabolism and enable visualization of their localization. Br-labeled fatty acid was also observed in phosphatidylethanolamine fractions (data not shown). Further studies are needed to precisely examine the distribution of each glycerophospholipid. Pulse-labeled Br-glycerophospholipids were likely divided into daughter cells via mitosis, and the strongest Br signals remained in the perinuclear region in daughter cells. The findings suggest that Br-glycerophospholipids function in daughter cells as well as mother cells. After cell division was halted due to contact inhibition, more Br appeared to be released from the cells. This suggested that cells either require Br-glycerophospholipids for cell proliferation or release Br-glycerophospholipids or digested one during metabolism, as seen by the changes in Br-PC and as suggested previously (25, 27).

### Limitation of the new method

Our results suggest that single-element labeling of fatty acids facilitated imaging and was more effective than labeling large molecules, such as fluorescent markers or proteins (12, 28). However, this system had a limitation. We measured the background level of Br in cells, which originated mostly from culture medium, as detected by SXFM (Supplemental Fig. 1), but Br was also measured in MeOH extractions using ICP-MS (Figs. 2*A* and 3*B*) and empty culture dishes. Based on additional experiments, we speculated that MeOH absorbed Br from the culture dishes. Br-free or low-Br culture dishes and media would thus be beneficial for reducing background levels; however, it is difficult to source Br-free equipment and media. Therefore, we recommend using the same equipment, culture media, and solutions throughout experiments to minimize and stabilize background levels, which is important for high-resolution imaging and quantitative analyses such as ICP-MS. We also need to consider the stability of Br-fatty acids in cells. We could not exclude the possibility that the Br-labeled compounds were digested or dehalogenated during cellular metabolism *in vivo.* However, bromine labeling has the advantages of direct binding to small molecules without the need for an additional binding motif (*e.g*., metal chelator domain), binding under mild conditions to retain biologic activity (29), and detecting less background distinguishable from endogenous unlabeled molecules by various measurement technologies, including SXFM. Considering these advantages, Br-labeled compounds were applied to PET imaging studies using animals, and PET images were successfully obtained (29, 30). The stability of cultured cells might be similar to PET, and the labeled fatty acids in the cells seemed sufficiently stable for imaging.

### Future applications of single-element labeling of fatty acids

Despite the above limitations, we established a novel procedure for imaging of intracellular fatty acids by single-element labeling combined with SXFM technology. Br signals observed by SXFM were validated as Br-fatty acids (Br-glycerophospholipids) using ICP-MS and LC-MS. Various applications, such as gene regulation of enzymes related to fatty acids, will reveal the details of their localization in cells, which would correspond to their functions. The visualization of special and temporal changes in microdomain or vesicular trafficking is another challenge. The application of this new technology to unsaturated fatty acids will enhance our understanding of the movement of eicosanoids or other polyunsaturated fatty acid-derived lipid mediators in various physiology or pathology (*e.g*., inflammation, neurodegenerative diseases, and cardiovascular diseases) (9, 31). SXFM can now be used to image mitochondria at the organelle level (18). Moreover, greater sensitivity due to the use of higher numbers of photons produced by a synchrotron facility and higher resolution due to modification of the X-ray focusing system are expected in the near future (22, 32, 33). Single-element labeling of fatty acids combined with X-ray fluorescence microscopy, in addition to chemical and biochemical procedures, should facilitate investigation of the intracellular dynamics of lipids.

## Abbreviations

ER: endoplasmic reticulum
EtOAc: ethyl acetate
HRMS: high-resolution mass spectrometry
ICP-MS: inductively coupled plasma mass spectrometry
IR: infrared
LC-MS: liquid chromatography mass spectrometry
MVE: multivesicular endosome
NMR: nuclear magnetic resonance
PA: palmitic acid
PC: phosphatidylcholine
PET: positron emission tomography
SA: stearic acid
SXFM: scanning X-ray fluorescence microscopy
TMAH: tetramethylammonium hydroxide
